# Bifrontal solitary fibrous tumor of the meninges

**DOI:** 10.4103/2152-7806.66852

**Published:** 2010-07-29

**Authors:** Michael Benoit, Robert-Charles Janzer, Luca Regli

**Affiliations:** Neurosurgery Department, Centre Hospitalier Universitaire Vaudois (CHUV), Rue du Bugnon 46, Switzerland; 1 University Institute of Pathology, Centre Hospitalier Universitaire Vaudois (CHUV), Rue du Bugnon 25, 1011 Lausanne, Switzerland; 2 Rudolf Magnus Institute of Neuroscience, Department of Neurosurgery, University Medical Center Utrecht, Heidelberglaan 100, 3508 GA Utrecht, Netherlands

**Keywords:** CNS tumors, meningeal tumors, p53, solitary fibrous tumor

## Abstract

**Background:**

We report the case of a bifrontal solitary fibrous tumor (SFT) arising from the meninges. The points of interest in this case report are the particular imaging appearance, the immunohistochemical findings and the surgical features.

**Case Description:**

A 53-year-old Caucasian male presented with a 1-year history of behavioral changes, attention disorders and anterograde memory disorders. Magnetic resonance imaging revealed a bifrontal heterogeneous lesion attached to the anterior falx cerebri with a prominent multicompartmental cystic part. The patient underwent craniotomy for a sub-total resection of the tumor. At surgery, the multicystic component was highly vascularized and encased the anterior cerebral arteries. Neuropathological findings were consistent with a solitary fibrous tumor. Despite the absence of malignant features, there was a focal expression of p53.

**Conclusion:**

SFT is a pathological entity with specific immunohistochemical features; it has frequently been misdiagnosed in the past. The multicystic imaging appearance of this SFT and the particular p53 immunohistochemical staining are features that should be added to the growing data on intracranial SFTs. The surgical features described (high vascularization and partial vessel encasement) may help improve surgical planning.

## INTRODUCTION

Solitary fibrous tumors (SFTs) were first described in the visceral pleura by Klemperer and Rabin [14] but have been subsequently reported to occur in almost every site of the body.[5,9,16,20,22,25,26,28,29]

SFTs in the CNS were first reported in 1996 by Carneiro *et al*.,[6] and since then fewer than 100 cases have been reported. SFTs can be found in various intracranial and spinal locations, but they seem to show a preference for the posterior fossa (26%) and the spinal region (25%).[23]

The subject of this case report is an unusual dura -based SFT arising from the falx cerebri, invading both frontal lobes. What makes this report interesting is the particular imaging appearance due to a large multicystic component, and the immunohistochemical staining. In addition, the highly vascularized structures and the arterial encasement are important features of this SFT that help improve surgical planning.

## CASE DESCRIPTION

The patient was a 53-year-old right-handed Caucasian male who presented with a 1-year history of behavioral changes characterized by a severe frontal syndrome (apathy, abulia, lack of energy, anosognosia, familiarity and disinhibition), as well as attention disorders and anterograde memory deficits. He also described persistent bifrontal headaches for the same period of time, which were relieved by simple analgesia.

General physical examination was unremarkable, although recent-onset obesity (BMI, 34) was noted.

Neurological examination revealed a cooperative and oriented patient with no cranial nerve deficiencies. No focal sensory and motor disabilities were noted, except for a right plantar reflex in extension.

### Imaging studies

**Figure 1:**
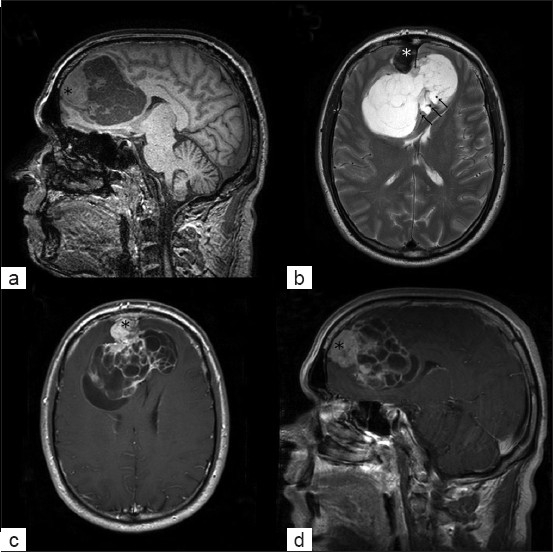
Magnetic resonance imaging of a large frontal interhemispheric heterogeneous lesion; (a) sagittal T1-weighted scan; (b) axial and coronal T2-weighted images showing the important mass-effect and the partial inclusion of both anterior cerebral arteries and their branches in the tumor (arrows); (c) and (d) axial and sagittal gadolinium-enhanced T1-weighted images showing both the anterior solid (asterisk) and the multicystic components of the lesion with the homogenous enhancement of the solid part, as well as the enhancement of the thick cystic walls

The MRI examination revealed a large bifrontal extra-axial lesion with an attachment to the anterior falx cerebri. The lesion was heterogeneous, presenting two distinct aspects: a smaller, fleshy, nodular part attached to the meninges (23 mm in diameter) and a large (41 × 87 × 68 mm) multi-compartmented cystic part with thick septa and fleshy parts. The lesion was markedly hyperintense on T2 images and had two distinct contrast enhancement patterns on T1 images, with a homogeneous enhancement of the fleshy part and heterogeneous enhancement limited to the septa in the cystic part [Figure 1]. The lesion exerted an important mass-effect on the corpus callosum and on the anterior horns of the lateral ventricles. No peri-lesional edema was noted.

An important point to mention is that the lesion encased both A2 segments of the anterior cerebral arteries [ACAs] and their branches.

### Surgery

The patient underwent a bifrontal craniotomy and a subtotal resection of the tumor. The preoperative plan was to access the tumor interhemispherically, remove the solid, fleshy part and then resect the multicystic part. The encasement of both ACAs in the tumor appeared to present a major challenge.

The tumor was firm and relatively avascular in its anterior nodular part. Microsurgical dissection identified a clear arachnoidal plane. Resection of the anterior nodular part was easily performed. Once the multicystic portion was reached, the arachnoidal plane of dissection disappeared and despite meticulous microsurgical dissection, the plan had to be subpial. The intralesional consistency also changed once the multicystic component was reached. The multiple septa were thick and extremely vascular. Hemostasis was tedious despite abundant bipolar electrocoagulation. The surgical impression was similar to a hemangiopericytoma. In line with the absent tumor-brain interface, the ACAs were adherent to the tumor capsule and encased within the tumor in its multicystic part. Tumor resection was subtotal (99%), with only tiny tumor remnants on the anterior cerebral artery branches.

### Outcome

The postoperative recovery was uneventful, and the patient showed complete remission of headaches and normalization of behavioral deficits. He returned to normal weight and was able to return to work.

**Figure 2:**
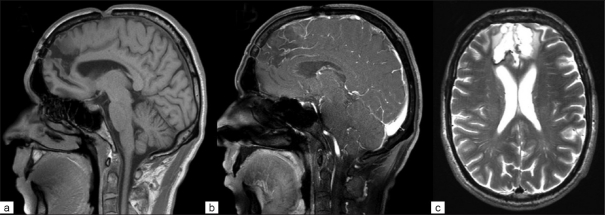
Postoperative magnetic resonance imaging showing no signs of tumor growth; (a) sagittal T1-weighted scan; (b) sagittal gadolinium-enhanced T1-weighted scan; (c) axial T2-weighted scan

At the 1-year follow-up, the patient was found to be symptom free. The MRI revealed only minimal tumoral residue on the right ACA, measuring 2 × 3 mm, with no evidence of growth, when compared to the condition at the 3-month follow-up [Figure 2].

### Histology

**Figure 3:**
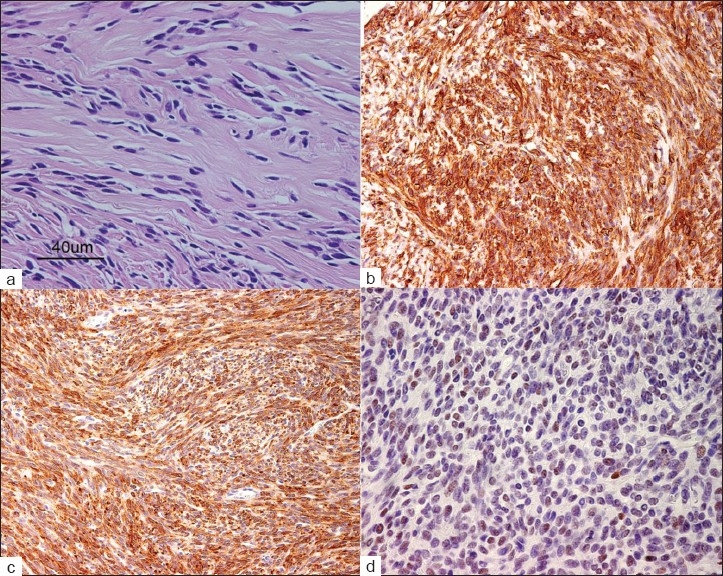
Histology and immunohistochemical profile of resected tumor (all original magnification at ×200; a+d at ×400): a) Tumor mostly containing spindle cells arranged in fascicles. Hematoxilyn and eosin staining coloration; b) Expression of CD34 in most tumor cells; c) Expression of Bcl-2 in most tumor cells; d) Nuclear expression of p53 in about half of the tumor cells

The compact tumor showed irregular borders with invasion of the dura and the subarachnoid space but no signs of brain invasion. The tumor was mostly composed of spindle-shaped cells disposed in a fascicular arrangement and separated by prominent eosinophilic bands of collagen [Figure 3a]. A minor component was compact with a diffuse and syncytial growth pattern and no collagen bundles. The vascular stroma of the tumor was rich. On histopathological examination, the wall of a small arterial branch of the ACA that was sacrificed did not show tumoral invasion.

There was strong cytoplasmic expression of vimentin, CD34, Bcl-2, CD99; and a focal nuclear expression of the progesterone receptor. Stainings for Epithelial Membrane Antigen (EMA), cytokeratins, protein S-100, CD117 and estrogen receptor were negative. The proliferation index indicated by an MIB-1 staining was low (less than 1%), and there were no more than 2 mitoses per 10 high-power fields. These morphological and immunohistochemical findings are in keeping with an SFT [Table 1 and Figure 3]. Of relevance is a focal positive reaction to p53.

**Table 1:** Immunohistochemical findings in SFTs in the literature and our patient

	Literature features	Features of our case	Meningiomas	HPCs
p53	−	+ Focally	+ Most cases	+
CD34	++	+	+ in 15% of cases	+ in 40% of cases
CD99	+	+	−	+
Bol-2	++	+	−	+/–
Vimentin	++	+	+	−
Progesterone R.	+ In some cases	+ Focally	+	−
EMA	−	−	+	−
Protein S-100	−	−	+	−
CD117	−	−	−	−

Features of meningiomas and hemangiopericytomas (HPCs) are added to allow comparison

## DISCUSSION

Solitary fibrous tumor (SFT) is an uncommon spindle cell tumor that typically arises from the visceral pleura.[7,14] The CNS SFT is its dura-based counterpart which has been classified in the World Health Organization. classification of tumors of the nervous system as a mesenchymal, non-meningothelial tumor.[11] The mesenchymal histopathogenic origin has been confirmed and accepted through immunohistochemical, ultrastructural and cell culture evidence.[1,3,9,21] Further evidence to support its mesenchymal nature is based on the following: the strong CD34 reactivity of the cells, the absence of mesothelial structures and the negative immunoreactivity to epithelial markers.[8,24]

The imaging characteristics described in this case report are quite atypical compared to the classical features of SFTs reported in the literature. Typically, SFTs present themselves as well-defined masses with low signal intensity on T2-weighted images and homogeneous enhancement on post-contrast T1-weighted images, leading to an initial radiographic impression of the more common meningiomas.[7,19,23] Intracranial SFTs tend to show a dural attachment; contrary to the spinal SFTs, which tend to show no dural attachment, despite their intradural location. Cases have been reported in deep locations such as the basal ganglia or ventricle, regardless of the proximity of the meninges.[12,15] The presence of two distinct consistencies within the same tumor is exceptional. We have described, on the one hand, a solid part presenting with typical characteristics; and on the other hand, a multicystic, multiloculated part with thick septa, which is more atypical. The multicystic part broadened the list of the differential diagnoses considerably, including atypical meningioma, cystic chondroma, chondrosarcoma and parasitic lesion (ecchinococosis). To our knowledge, this feature has not been reported before and should be added to the growing knowledge about the imaging appearances of SFTs in the CNS.

SFTs are radiologically and macroscopically indistinguishable from meningiomas but are clearly distinguished by histology and immunohistochemistry.[7,21]

Histologically, SFTs are tumors composed of spindle cells growing in fascicles separated by thick collagen bundles. More compact areas or irregular arrangements may be present. The vascular stroma may resemble the stag-horn pattern seen in hemangiopericytoma.[4,19] Our case shows an interesting correlation between the classical histological pattern and the macroscopic features. The “feeling-behavior” during surgery was similar to a hemangiopericytoma, hence confirming the rich vascularization seen on microscopic structure.

Immunohistochemically, SFTs are typically positive for vimentin, CD34, CD99 and Bcl-2. Protein S-100, EMA and CD117 are usually negative; as well as vascular, neural crest and muscle immunological markers also are negative.[6,7,10,17,19,24,25] The immunohistochemical pattern in our case is in accordance with these findings. Expression of the progesterone receptor, although typically seen in meningiomas, is also seen in SFTs.[2,8] However, its prognostic value has still to be established, particularly in intracerebral cases.

Expression of p53 has only been reported in SFTs located outside the CNS and in SFTs with histologically malignant features.[27] In this case, despite p53 expression, there were no histological signs of malignancy, such as nuclear pleomorphism, high mitotic index or local brain invasion. The prognostic value of p53 expression has yet to be determined. In our patient, there was no progression in size of the minimal residual at the 12-month follow-up.

SFTs are considered as benign tumors with a slow, indolent and nonaggressive course. Recurrence and metastasis are rare.[7,11,18] Four examples of clinically and histologically malignant SFTs have been reported,[7,13,23] presenting with the following features: increased number of mitotic figures (more than 4 mitoses per 10 high-power fields), hypercellularity, high proliferation index and nuclear pleomorphism. If the tumor is completely resected, the above-mentioned histological findings do not seem to be predictive of malignant behavior.[6] Therefore, the most important prognostic marker of SFTs is the extent of surgical resection rather than histologic appearance. Local recurrences have been reported, but only when an incomplete resection was achieved.[23] From this perspective, complete surgical treatment seems to be sufficient in most cases, and it would seem that there is no need for post-surgery adjuvant treatments such as chemotherapy or radiotherapy in the management of SFTs.[6] However, due to the short follow-up period of the reported cases and their small numbers in various reports, there is no consensus about the recommended post-surgical management of these tumors.

## CONCLUSION

SFTs represent a unique pathological entity that is more often recognized today due to clear immunohistochemical markers. Prior to the development of these markers, these tumors were often misdiagnosed. Until more data is gathered on the management of SFTs, the current literature suggests complete resection of the tumor and careful radiological follow-up as the optimal treatment program for all patients with SFTs. It should be kept in mind, however, that recurrence and metastasis are possible; even if the postoperative course is benign, as in most cases.

This case report is of interest because of the multicystic appearance, which adds new imaging features of CNS SFTs. It also emphasizes the importance of careful pre-surgical planning, because of the “hemangiopericytoma-like” vascularization pattern of the tumor and possible encasement in the tumor of arterial branches “*en passage*.” Finally, it also reports the positive p53 staining inside the CNS without malignant histological features.
